# Ultra-processed foods and obesity and adiposity parameters among children and adolescents: a systematic review

**DOI:** 10.1007/s00394-022-02873-4

**Published:** 2022-03-24

**Authors:** Ramona De Amicis, Sara Paola Mambrini, Marta Pellizzari, Andrea Foppiani, Simona Bertoli, Alberto Battezzati, Alessandro Leone

**Affiliations:** 1 International Center for the Assessment of Nutritional Status (ICANS), Department of Food, Environmental and Nutritional Sciences (DeFENS), University of Milan, Milan, Italy; 2 Division of Nutritional Rehabilitation, IRCCS Istituto Auxologico Italiano, Piancavallo, Italy; 3 Lab of Nutrition and Obesity Research, Istituto Auxologico Italiano, IRCCS, Milan, Italy

**Keywords:** Ultra-processed food, Obesity, Body fat, Abdominal fat, Children, Adolescents

## Abstract

**Purpose:**

According to the NOVA classification, ultra-processed foods are products made through physical, biological and chemical processes and typically with multiple ingredients and additives, in which whole foods are mostly or entirely absent. From a nutritional point of view, they are typically energy-dense foods high in fat, sugar, and salt and low in fiber. The association between the consumption of ultra-processed food and obesity and adiposity measurements has been established in adults. However, the situation remains unclear in children and adolescents.

**Methods:**

We carried out a systematic review, in which we summarize observational studies investigating the association between the consumption of ultra-processed food, as defined by NOVA classification, and obesity and adiposity parameters among children and adolescents. A literature search was performed using PUBMED and Web of Science databases for relevant articles published prior to May 2021.

**Results:**

Ten studies, five longitudinal and five cross-sectional, mainly conducted in Brazil, were included in this review. Four longitudinal studies in children with a follow-up longer than 4 years found a positive association between the consumption of ultra-processed food and obesity and adiposity parameters, whereas cross-sectional studies failed to find an association.

**Conclusion:**

These data suggest that a consistent intake of ultra-processed foods over time is needed to impact nutritional status and body composition of children and adolescents. Further well-designed prospective studies worldwide are needed to confirm these findings considering country-related differences in dietary habits and food production technologies.

## Introduction

Childhood obesity is one of the most worldwide public health challenges of the twenty-first century [1]. Its worldwide prevalence has risen dramatically from just 4% in 1975 to just over 18% in 2016 [2]. In the USA, 19.3% of children and adolescents suffers of obesity [3], while in European countries the prevalence is between 9 and 13%, with Mediterranean countries having the highest rates [4, 5]. Being obese during childhood and adolescence results in a higher risk of contracting a chronic disease, and can have short- and long-term consequences [6]. In the short term, psychological problems, eating disorders, asthma, and musculoskeletal problems can occur [7]. Overweight or obese youth also develop increased metabolic risk, through dyslipidemia, type 2 diabetes mellitus, or cardiovascular problems [8]. In the long term, there are socio-environmental factors that often prolong the state of obesity into adolescence and adult life. If obesity persists, it can lead to the chronicity of these diseases, which can cause disability and an increased risk of premature death [6].

Obesity is generally defined as an excessive accumulation of adipose tissue that can compromise health [2]. Therefore, defining obesity requires a suitable measurement of body fat. The body mass index (BMI) is a broadly accepted measure of weight in relation to height. As BMI naturally changes with age, and it is different for boys and girls, it must be compared to the range of BMI seen for children of the same age and sex using international (e.g., WHO, CDC, IOTF) [9–11] or national growth charts. For example, after age 5 years, WHO defines excess of body weight as a BMI-for-age greater than 1 standard deviation and obesity a BMI greater than 2 standard deviations. However, BMI fails to distinguish between fat and fat‑free mass and may exaggerate obesity in large muscular children, hence the need to associate to the BMI, some measures of adiposity, such as waist circumference (WC), other circumferences and ratios, and measures of body composition [12].

The causes of obesity are complex and still not fully known. However, it is plausible that the condition is driven largely by environmental factors that influence dietary choices [1]. Of these, education and income seem to lead the way. Low levels of education and income lead to shopping at low-cost stores and increased access to noxious foods, consistently associated with higher risk of childhood obesity [13, 14]. Conversely, high socioeconomic status is becoming the primary determinant of obesity in adolescents due to more frequent media use and consequently sedentary lifestyles coupled with greater exposure to advertising of low nutritional quality and energy-dense foods [15] that look attractive, hyper-palatable, cheap and ready to eat [16–19]. Moreover, the latest reports also predict how the COVID-19 pandemic could potentially amplify one of the most worrying trends in the WHO European Region [4]. Indeed, imposed social isolation seems to predispose to unhealthy nutritional behaviors [20] and results in increased consumption of processed foods, such as snacks, junk and ready-to-eat foods, compared to standard living conditions [21, 22].

According to the United States Department of Agriculture (USDA), processed food is a product that has been through a process that has altered its natural state. In the category of processed food, any food can be included except raw agricultural products. In this regard, researchers have developed methods to categorize foods according to the degree of processing, ranging from minimally to highly processed. Among the most used systems in literature, emerges the NOVA method, proposed by Monteiro et al. [19] in 2010, which classifies foods and beverages according to the purpose and degree of processing they are being exposed to. This classification identifies foods into four groups. Specifically: (1) unprocessed foods, such as edible parts of plants or animals, mushrooms, seaweed and water, or minimally processed foods, which are natural foods that have been treated to make them safe and suitable for storage, edible or more pleasant to consume; (2) processed culinary ingredients, such as oils, butter, lard, sugar and salt, designed to be combined with foods and make dishes palatable; (3) processed foods made mostly by adding salt, oil, sugar or other group 2 substances to group 1 foods, such as canned vegetables or legumes preserved in pickle, whole fruit stored in syrup, canned fish preserved in oil; (4) ultra-processed foods and beverages (UPF), which are made through physical, biological and chemical processes and typically with multiple ingredients and additives, after foods are separated from nature, and before being consumed or prepared as dishes and meals. They are: soft drinks, packaged sweet or savory snacks, mass-produced packaged breads and buns, processed meats, and pre-prepared frozen meals [19]. These last ones are created with low-cost ingredients to be highly profitable, attractive and convenient (long shelf-life and ready to eat). The literature suggests that such foods, with their poor nutritional quality and high energy density, are able to alter hunger and satiety mechanisms by promoting excessive energy consumption [18, 23].

Several prospective cohorts and clinical trials in the adult population linked the high UPF consumption with the risk of obesity [24–26], weight gain [27] and a greater accumulation of total and visceral fat [28]. Especially, a recent meta-analysis in adults found that the consumption of UPF was associated with a 36% and 51% significant higher risk of overweight and obesity, respectively [29]. Concerning childhood and adolescence, UPF consumption is phenomenon of major importance that is rapidly growing. Children’s diets in the USA have shifted to contain about two-thirds of daily calorie consumed from UPF, contributing to high levels of body fat in children [30]. In Mediterranean countries, their consumption is more modest, but increasing. In Italy, a telephone survey collected between 2010 and 2013, shows a percentage of daily energy from UPF amounting to a quarter of total energy in children and adolescents [31]. In Spain, 32.2% of children’s total energy intake came from UPF, showing an inverse association between adherence to the traditional Mediterranean diet and consumption of UPF [32]. Nevertheless, the association between their consumption and obesity and adipose tissue in childhood and adolescence remains poorly understood.

Therefore, the present systematic review aims to synthesize the current available literature by further exploring the association between UPF consumption, obesity and adiposity parameters among children and adolescents to boost new primary prevention policies to regulate UPF consumption and promoting healthier nutritional status also in childhood and adolescence.

## Materials and methods

### Search strategy

This review has been performed according to the Preferred Reporting Items for Systematic Reviews and Meta-Analyses (PRISMA) guidelines. The electronic literature was searched in 2 databases, including PubMed and Web of Science, for articles published between 2010, when the NOVA classification system was developed, and May 2021. The full electronic search was conducted using the following keywords and combinations: (‘ultra-processed food’ OR ‘ultraprocessed food’) AND (‘obesity’ OR ‘BMI’ OR ‘body fat’ OR ‘adipose tissue’ OR ‘fat mass’ OR ‘fat free mass’ OR ‘muscle mass’ OR ‘body composition’ OR ‘waist circumference’) AND (‘children’ OR ‘adolescents).

### Study selection, inclusion and exclusion criteria

After exclusion of duplicates, two independent authors reviewed and selected relevant articles based on title and abstract. Once selected the relevant articles to this review, they evaluated the eligibility of the selected articles based on the following inclusion and exclusion criteria. To be eligible, studies had to meet these inclusion criteria: be original article, conducted in children and/or adolescents, written in English, using an observational study design (e.g., cross-sectional, cohort, case–control designs), using the NOVA food classification system for UPF, investigating the association between consumption of UPF and obesity and adiposity parameters in children and/or adolescents. Studies were excluded if they were reviews or were studies that only evaluated the intake of one food category included in the UPF definition (e.g., sugar-sweetened beverages or processed meat) or did not assess the direct consumption of UPF (e.g., household availability or purchase of UPF). When the two reviewers disagreed, a third reviewer was involved and decided whether the articles should be kept or excluded. The third reviewer judged the relevance of two articles on which the two reviewers disagreed and decided to exclude them both.

### Data extraction

The selected articles were fully analyzed to extract the following information: authors, publication year, country, study design, sample size, sample age, method used for the assessment of dietary habits, details about the outcomes (what data have been collected, tools and results), confounders used to adjust the analysis, and main results.

### Quality assessment

Critical Appraisal Checklists for Cross-Sectional and Cohort Studies proposed by the Joanna Briggs Institute were used to assess the methodological validity of the selected studies [33]. The checklists consisted of 8 and 11 question items, respectively, assessing several domains: population characteristics, exposure, confounders, outcomes, follow-up and statistical analysis. Four possible responses are provided for each item: yes, no, unclear or not applicable. Based on the answers, the checklists provide an overall critical appraisal to include or exclude a study. Studies that answered at least half of the questions with “yes” answers—for example, 4 of 8 items in cross-sectional studies and 6 of 11 items in cohort studies—were considered as having acceptable quality to be included in this systematic review [34].

## Results

### Study characteristics

**Fig. 1:**
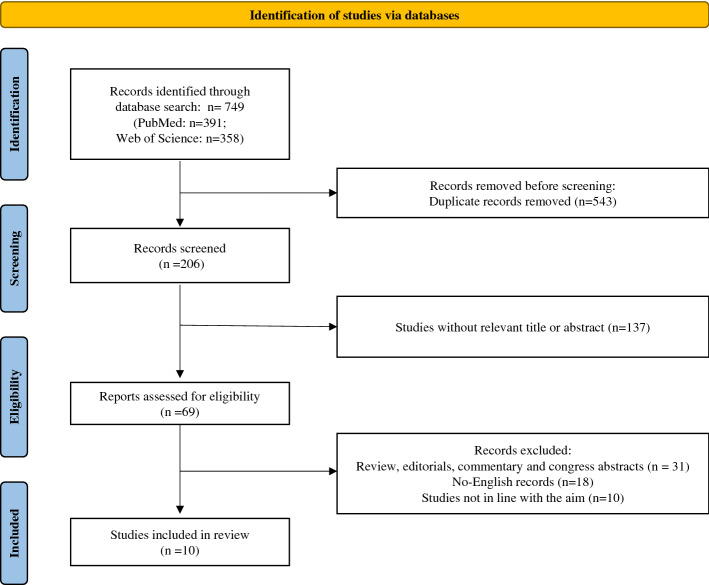
Flow diagram of study selection for systematic review

**Fig. 2:**
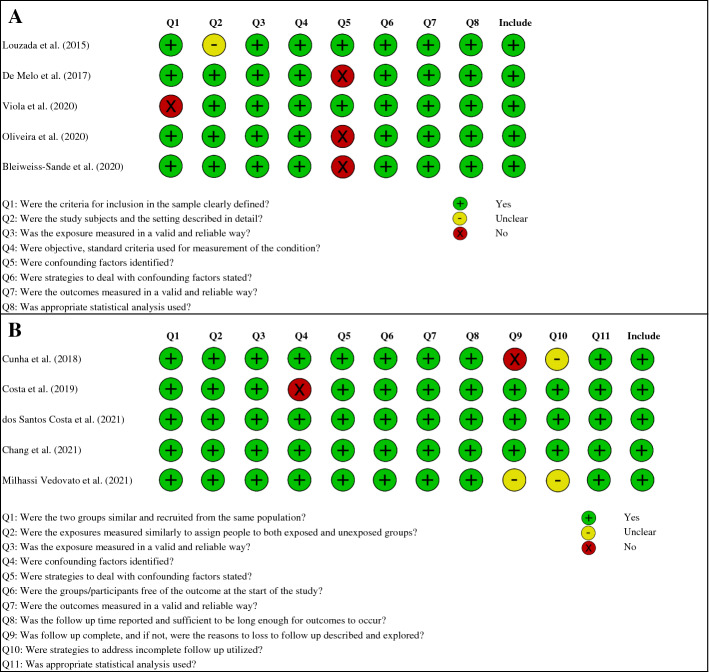
Critical Appraisal Checklist for cross-sectional (**A**) and cohort (**B**) studies

We initially retrieved a total of 749 potential articles published between 2010 and May 2021, including 391 from PubMed and 358 from Web of Science. After excluding 543 duplicates, we retained 206 articles. Next, we discarded 137 irrelevant articles based on title or abstract. The remaining 69 records were assessed for eligibility. We discarded 31 reviews, meta-analysis, editorials, commentary and congress abstracts, 18 articles not written in English and 10 articles not in line with the purpose of this review. In the end, a total of 10 studies were selected for the present review. The flowchart of the literature search and exclusion process is shown in Fig. 1. All studies included in this review underwent quality assessment as described above, and all were found to have overall sufficient methodological quality (Fig. 2).

Although the NOVA classification was defined in 2010, the first study investigating the association between UPF consumption and obesity and adiposity parameters in children and adolescents was published in 2015. The 10 studies [35–44] included a total of 24,281 children and adolescents with an age range of 4 to 20 years (Table 1). The sample size ranged from 131 to 9025 individuals. Six studies [38, 40–44] involved children (*n* = 13,938, age range 4–10 years) and four studies [35–37, 39] involved adolescents (*n* = 10,343, age range 10–20 years). Seven studies were conducted in Brazil [35–40, 42], and the remaining 3 studies were performed in Portugal [44], the United States [41], and England [43]. Five studies were cross-sectional [35, 36, 39–41] and five used longitudinal design [37, 38, 42–44]. All studies included children or adolescents of both sexes. To assess the UPF consumption, four articles used a food frequency questionnaire [36, 37, 39, 42], four studies used a 24-h recall on 2 or 3 non-consecutive days [35, 38, 40, 41], and two papers used a 1-, 2- or 3-day food dietary record [43, 44]. Consumption of UPF (exposure variable) was computed as follow: as percentage of energy contribution in the total energy intake (%UPF_energy_) in five studies [35, 38–41], as daily frequency of UPF consumption in two studies [36, 37], as percentage of weight contribution in the total daily food intake (%UPF_intake_) in one study [43], as absolute calories from UPF in one study [44], and as daily UPF intake in grams in one study [42].

**Table 1:** Summary of the selected studies that investigated the association between the UPF consumption and obesity and adiposity parameters in children and adolescents

Authors, year and country	Study design	Study population	Exposure variable and assessment	Outcomes and methods	Confounders	Main results
Louzada et al. (2015), Brazil [21]	Cross-sectional	7534 adolescents aged 10–19 years	%UPF_eneergy_; quintiles Two 24-h dietary recalls on 2 non-consecutive days	BMI; risk of obesity (BMI-for-age *z* score ≥ + 2); risk of weight excess (BMI-for-age *z* score ≥ + 1)	Age, sex, race, region, urban status, smoking, physical activity, education, per capita household income, consumption of fruits, vegetables and beans, interaction between sex and income	Adolescents in the upper quintile of UPF consumption had a BMI (mean difference 0.84 kg/m^2^, 95% CI − 0.16, 1.85; *P*_trend_ = 0.08) and the risk for excess weight (OR = 1.52, 95% CI 0.75, 3.07; P_trend_ = 0.250) similar to adolescents in the first quintile of UPF consumption. A positive significant trend was observed between the UPF consumption and the risk for obesity, but adolescents in the upper quintile had the same risk for obesity of those in the lower quintile (OR = 2.74, 95% CI 0.78, 9.60; P_trend_ = 0.05)
Melo et al. (2017), Brazil [22]	Cross-sectional	249 adolescents aged 14–19 years; 45.2% boys and 55.8% girls	Daily frequency of UPF consumption; group 1 (less than weekly) and group 2 (weekly or more) Food frequency questionnaire (84-food items)	Excess of body weight (BMI-for-age *z* score > + 1) and high WC identified using sex- and age-specific cutoff values	Age and sex	No association was found between the frequency of consumption of UPF and the excess of body weight (PR = 0.76, 95% CI 0.47, 1.22; *P* = 0.25) and high WC (PR = 0.94, 95% CI 0.51, 1.72, *P* = 0.85)
Cunha et al. (2018), Brazil [23]	Longitudinal prospective cohort	1035 adolescents aged 14–20 years; 46% boys and 54% girls	Daily frequency of UPF consumption; quartiles Food frequency questionnaire (FFQ: 72 food items)	BMI at baseline and at 1- and 2-year follow-up; %BF by bioelectric impedance at baseline and at 2-year follow-up	Type of school (public or private), sex, physical activity levels, and underreporting (a dummy variable for energy intake below the 10th percentile)	An inverse association was found between the frequency of UPF consumption and BMI, both at baseline and at 1-year follow-up, and %BF at 2-year follow-up
Costa et al. (2019), Brazil [24]	Longitudinal	315 children aged 4 years; 57% boys and 43% girls	%UPF_eneergy_; continuous Two 24-h dietary recalls on 2 non-consecutive days at baseline and four years later	BMI, WC, WHtR and skinfolds sum (tricipital and subscapular) changes from 4 to 8 years old	Group status in the early phase (intervention and control), mother’s pre-pregnancy BMI, sex, birth weight, breast-feeding, family income, maternal schooling and total screen	UPF consumption at 4 years old was associated with increased WC change from preschool to school age (*β* = 0.07, 95% CI 0.01, 0.14; *P* = 0.030). No association was found between the UPF consumption and BMI (*β* = 0.00, 95% CI − 0.02, 0.01; *P* = 0.569), WHtR (*β* = 0.00, 95% CI 0.00, 0.00; *P* = 0.108) and skinfolds sum (*β* = 0.05, 95% CI − 0.04, 0.15; *P* = 0.282) changes
Viola et al. (2020), Brazil [25]	Cross-sectional	1525 adolescents aged 18–19 years; 47.1% boys and 52.9% girls	%UPF_eneergy_; continuous Food frequency questionnaire (FFQ: 106 food items)	BMI, WC, %BF by air displacement plethysmography (BODPOD), muscle mass, LMI and android fat by DEXA	Sex, education, socioeconomic status, total daily energy intake, dieting to lose weight, physical activity, consumption of alcoholic beverages, smoking, and sleeping hours	Inverse relationship between the consumption of UPF and BMI (*β* = − 0.01 kg/m^2^, 95% CI − 0.03, − 0.01; *P* = 0.021), muscle mass (*β* = − 0.04 kg, 95% CI − 0.06, − 0.02; *P* < 0.001), and LMI (*β* = − 0.01 kg/m^2^, 95% CI − 0.02, − 0.01; *P* < 0.001). No association was found between the consumption of UPF and %BF, WC and kg of android fat
Oliveira et al. (2020), Brazil [26]	Cross-sectional	164 children aged 7–10 years; 40.9% boys and 59.1% girls	%UPF_eneergy_; continuous Three 24-h recalls carried out on non-consecutive days (one of them on weekend)	BMI, WC and WHtR	Age, sex and total caloric intake	No significant association was found between the UPF consumption and BMI (*β* = − 0.004, 95% CI − 0.057, 0.048), WC (*β* = − 0.037, 95% CI − 0.167, 0.092) and WHtR (*β* = 0.001, 95% CI − 0.001, 0.001)
Bleiweiss-Sande et al. (2020), United States [27]	Cross-sectional	131 children aged 6–12 years from low-income communities; 58% boys and 42% girls	%UPF_eneergy_; continuous Three 24-h recalls carried out on non-consecutive days (one of them on weekend)	BMI-for-age *z* score	Sex, days of National School Breakfast and National School Lunch Program participation	No association was found between the UPF consumption and BMI-for-age *z* score (*β* = 0.0006; 95% CI − 0.0068, 0.0080; *P* = 0.87)
Costa et al. (2021), Brazil [28]	Longitudinal prospective cohort	3128 children aged 6 years; 51.8% boys and 48.2% girls	Daily intake of UPF (grams) from 6 to 11 years of age; continuous Food frequency questionnaire (54-food items and 88-food items at 6- and 11-year follow-ups	FMI from 6 to 11 years of age. Body fat was measured by air displacement plethysmography (BODPOD)	Model 1: Maternal age, schooling and skin color, participant sex, birthweight, time spent watching TV, daily energy intake: expenditure ratio Model 2: Model 1 + consumption of foods other than UPF (g/day) Model 3: Model 1 + daily energy intake	UPF consumption was associated with FMI change between 6 and 11 years of age. In model 1, a daily increase of 100 g in the contribution from UPF was associated with a FMI gain of 0.09 kg/m^2^ (95% CI 0.07, 0.10; *P* < 0.001). In model 2, a daily increase of 100 g in the contribution from UPF was associated with a FMI gain of 0.14 kg/m^2^ (95% CI 0.13, 0.15; *P* < 0.001). In model 3, a daily increase of 100 g in the contribution from UPF was associated with a FMI gain of 0.05 kg/m^2^ (95% CI 0.04, 0.06; *P* < 0.001)
Chang et al. (2021), England [29]	Longitudinal prospective cohort	9025 children: 80.5% (*n* = 7264) aged 7 years, 16.8% (*n* = 1519) aged 10 years of age, and 7.2% (*n* = 242) aged 13 years; 50.3% boys and 49.7% girls	%UPF_intake_; quintiles 3-day food diary (79.0% of cases), 2-day food diary (13.0% of cases) and 1-day food dairy (8.0% of cases)	Primary outcomes: BMI, FMI, LMI and %BF measured by DEXA from recruitment to 24 years old Secondary outcomes: BMI-for-age *z* score, weight, WC, fat mass and lean mass by DEXA from recruitment to 24 years old	Child's sex, ethnicity, birth weight, physical activity, quintiles of Index of Multiple Deprivation and total energy intake, and mother's pre-pregnancy BMI, marital status, education and socioeconomic status	Primary outcomes: At baseline, children in the upper quintile of UPF consumption had higher mean values for FMI (+ 0.27 kg/m^2^, 95% CI 0.09, 0.45) and %BF (+ 1.47%, 95% CI 0.81, 2.13), but not for BMI and LMI, compared to children in the first quintile. Growth trajectories for BMI and FMI were significantly higher in the fifth quintile compared to the first quintile by an additional 0.06 kg/m^2^ (95% CI 0.04, 0.08) and 0.03 kg/m^2^ (95% CI 0.01, 0.05) per year, respectively Secondary outcomes: At baseline, children in the upper quintile of UPF consumption had higher mean values for FM (+ 0.51 kg, 95% CI 0.08, 0.93), but not for body weight, WC, BMI-for-age *z* score and lean mass, compared to children in the first quintile. Growth trajectories for body weight, WC, BMI-for-age *z* score and FM were significantly higher in the fifth quintile compared to the first quintile by an additional 0.20 kg (95% CI 0.11, 0.28), 0.17 cm (95% CI 0.11, 0.22), 0.01 *z* score (95% CI 0.003, 0.01) and 0.15 kg (95% CI 0.08, 0.21) per year, respectively
Vedovato et al. (2021), Portugal [30]	Longitudinal prospective cohort	1175 children aged 4 years; 52% boys and 48% girls	Calories from UPF (kcal/day); continuous 2-day or 3-day food dietary records (1 or 2 weekdays and 1 weekend day) at 4 and 7 years of age	BMI-for-age *z* score at 10 years	Maternal age, education and BMI before pregnancy, exclusive breast-feeding for the first 6 months, parental concern on eating behaviors, child BMI *z* score, practice of physical exercise and daily screen time at 4 years of age	The UPF consumption at 4 years old was significantly associated with BMI-for-age *z* score at age 10 (*β* = 0·028; 95% CI 0·006, 0·051 every 100 kcal/day from UPF). No association was found between the UPF consumption at 7 years old was and BMI-for-age *z* score at age 10

*%UPF*_*energy*_ proportion of energy from ultra-processed food to total energy intake, *%UPF*_*eneergy*_ proportion of intake from ultra-processed food to total energy intake, *WC* waist circumference, *WHtR* waist-to-height ratio, *WHR* waist-to-hip ratio, %*BF* percentage of body fat, *LMI* lean mass index, *FMI* fat mass index

### Association between UPF consumption and BMI

The association between UPF consumption and BMI was investigated in eight studies [35, 37–41, 43, 44]: in five studies the outcome was the BMI not standardized for sex and age [35, 37–40] and in three studies the outcome was BMI-for-age *z* score [41, 43, 44]. Four studies were cross-sectional [35, 39–41] and four were prospective [37, 38, 43, 44]. Five study enrolled children [38, 40, 41, 43, 44] and three enrolled adolescents [35, 37, 39]. Two prospective studies reported a positive association between UPF consumption and BMI-for-age *z* score [43, 44]. In a cohort of 9025 7-year-old children, Chang et al. [43] did not observe an association between baseline UPF consumption, calculated as UPF_intake_, and baseline BMI and BMI-for-age *z* score values. However, assessing growth trajectories up to the age of 24 years, BMI and BMI-for-age *z* scores increased more in children in the top quintile of UPF consumption than in children in the bottom quintile. In detail, these increased by 0.06 kg/m^2^ (95% CI 0.04, 0.08) and 0.01 *z* score (95% CI 0.003, 0.01) more per year, respectively. In another cohort of 1175 4-years-old children, Vedovato et al. [44] observed that for every 100 kcal from UPF, consumed at age 4 years, the BMI-for-age *z* score at 10 years increased by 0.028 *z* score (95% CI 0.006, 0.051). No association was found between UPF consumption at age 4 years and BMI-for-age *z* score at 7 years of age. Contrary, two further studies, one prospective and one cross-sectional, found a negative association between UPF consumption and the BMI not-standardized for sex and age [37, 39]. In the first study, involving 1035 adolescents aged 14–20 years, Cunha et al. [37] found that adolescents in the upper quartile of UPF consumption, expressed as daily frequency of consumption, had lower BMI, both at baseline and after 1 year of follow-up, than adolescents in the first quartile. Similarly, in the cross-sectional study conducted by Viola et al. [39] on a sample of 1525 adolescents aged 18–19 years, as the %UPF_energy_ increased, BMI decreased to the extent of 0.01 kg/m^2^ for each 1 point of %UPF_energy_. Finally, three cross-sectional studies [35, 40, 41] and one prospective study with a 4-year follow-up [38] did not find an association between UPF consumption and BMI. Two of the three cross-sectional studies and the prospective study used the BMI not-standardized for sex and age as outcome, whereas the third cross-sectional study used the BMI-for-age *z* score.

### Association between UPF consumption and risk of obesity and excess weight

Two studies assessed the association between the UPF consumption and the risk of obesity and excess weight, defined as BMI-for-age *z* score >  + 2 and BMI-for-age *z* score >  + 1, respectively [35, 36]. Both studies were cross-sectional and, overall, involved 7783 adolescents. In the first study, Louzada et al. [35] assessed the food consumption of 7534 adolescents aged 10–19 years and observed a non-significant higher risk of obesity (OR = 2.74; 95% CI 0.78, 9.60) and excess weight (OR = 1.52; 95% CI 0.75, 3.07) in adolescents in the upper quintile of UPF consumption (≥ 52% of daily energy intake from UPF) compared to those in lower quintile (< 17% of daily energy intake from UPF). In the second study, Melo et al. [36] recruited 249 adolescents aged 14–19 years and did not observe any association between the frequency of consumption of UPF and the risk of excess weight. Adolescents consuming UPF weekly were as likely to be overweight as adolescents who consumed UPF less frequently (PR = 0.76; 95% CI 0.47, 1.22).

### Association between UPF consumption and abdominal obesity

Five studies assessed the association between UPF consumption and abdominal obesity, measuring WC, waist-to-height ratio (WHtR) or waist-to-hip ratio (WHR) [36, 38–40, 43]. Of the five studies, three were cross-sectional [36, 39, 40] and two were prospective [38, 43]. Three studies were conducted on children [38, 40, 43] and two on adolescents [36, 39]. Measurements of abdominal obesity were found significantly associated with the UPF consumption only in prospective studies [38, 43]. Costa et al. [38] conducted a longitudinal study involving 307 4-year-old children, in which they assessed UPF consumption, and WC and WHtR. After a follow-up of 4 years, they evaluated WC and WHtR changes in relation to baseline UPF consumption, and observed that for every 10% increase in %UPF_energy_, WC at 8 years was increased by 0.7 cm (*β* = 0.07; 95% CI 0.01, 0.13) compared with baseline, while no association was found with WHtR change. In a second longitudinal study, Chang et al. [43] observed that WC, over the period from 7 to 24 years of age, increased by an additional 0.17 cm (95% CI 0.11, 0.22) per year in children in the highest quintile of UPF consumption compared with children in the lowest quintile of UPF consumption. Differently, in three cross-sectional studies, two involving adolescents [36, 39] and one involving children [40], the UPF consumption was not associated with the measurements of abdominal obesity.

### Association between UPF consumption and body fat

Five studies [37–39, 42, 43] assessed the association between the consumption of UPF and body fat. Four studies were prospective [37, 38, 42, 43] and one cross-sectional [39]. Three studies were conducted on children [38, 42, 43] and two on adolescents [37, 39]. To assess body fat, one study used the sum of tricipital and subscapular skinfolds [38], one used bioelectric impedance [37], and three used gold-standard methods, as BODPOD and DEXA [39, 42, 43]. Two prospective studies found a positive association between UPF consumption and body fat [42, 43]. Costa et al. [42] enrolled 3128 6-year-old children in whom they assessed dietary habits by a FFQ and body fat by BODPOD. After a 5-year follow-up period, these measurements were repeated and changes of UPF consumption (in grams) and fat mass index (FMI) were correlated. The authors found that a 100-g increase in UPF consumption from 6 to 11 years of age was associated with a FMI gain of 0.14 kg/m^2^ (95% CI 0.13, 0.15) over the same time period. The association was attenuated, although it remained significant, after controlling for total energy intake (*β* = 0.05 kg/m^2^, 95% CI 0.04, 0.06). Cheng et al. [43] observed that FMI, over the period from 7 to 24 years of age, increased by an additional 0.03 kg/m^2^ (95% CI 0.01, 0.05) per year in children in the highest quintile of UPF consumption compared with children in the lowest quintile of UPF consumption. Contrary to these findings, in a cohort of 1039 adolescents aged 14–20 years, Cunha et al. [37] reported an inverse association between baseline UPF consumption and body fat percent (%BF) measured by bioelectrical impedance two years after recruitment. In contrast with these results, a cross-sectional study involving 1525 adolescents [39], did not find an association between the consumption of UPF and android fat measured by DEXA and %BF measured by BODPOD. However, %UPF_energy_ was found inversely associated with muscle mass (*β* = − 0.04 kg, 95% CI − 0.06, − 0.02; *P* < 0.001) and LMI (*β* = − 0.01 kg/m^2^, 95% CI − 0.02, − 0.01) measured by DEXA. Finally, in a further longitudinal study [38], the consumption of UPF at preschool age was not a predictor of change in the sum of tricipital and subscapular skinfolds from preschool to school age.

## Discussion

This systematic review shows that the current documented evidence regarding the association between UPF consumption and obesity and adiposity parameters in children and adolescents is limited and heterogeneous. Nevertheless, four studies provided evidence that the UPF consumption was directly and positively associated with one or more weight and fat outcomes [38, 42–44]. These studies have common characteristics, namely the prospective design, the recruitment of children, and a follow-up time of at least 4 years. By contrast, both cross-sectional [35, 36, 39–41] and prospective studies with less than 4 years of follow-up [37] found no or inverse association between UPF consumption and parameters of obesity and adiposity. This suggests that a consistent intake of UPF over time is needed to impact nutritional status and body composition of children and adolescents. To our knowledge, this is the first systematic review to investigate the association between consumption of UPFs, defined according to an internationally recognized classification, such as the NOVA classification, and weight- and fat-related measures in children and adolescents. However, these data should be interpreted with caution because of the limited number of studies and high heterogeneity among them.

Several mechanisms have been proposed to describe the association between intake of ultra-processed foods and weight-related outcomes. UPF are, in general, more energy-dense, high in refined carbohydrates and saturated and trans-fatty acids, low in fiber, and contain added sugars and sodium [18]. In addition, high consumption of ultra-processed foods can reduce total energy expenditure because of the reduced thermic effect of the foods. An experimental study showed a 50% reduction in postprandial energy expenditure following consumption of ultra-processed foods, compared with unprocessed iso-caloric foods [45]. In addition, ultra-processed foods rich in refined carbohydrates and sugars may alter insulin levels and increase nutrient storage in adipose tissue [46]. In addition, the structural and physical properties of ultra-processed products, as well as the low fiber content, may alter satiety signaling, causing overconsumption [47]. Finally, consumption of ultra-processed foods may increase exposure to non-nutrients components, such as phthalates and bisphenol A [48]. These molecules are endocrine disruptors thought to be involved in the pathogenesis of obesity [49, 50]. However, it is well known that childhood and adolescence are phases of human life involving an increase in body tissues that results in an increase of energy expenditure and metabolic activity. This aspect may, to some extent, delay or offset the effect of UPF on weight and fat measures [29], and may be the reason for the lack of a positive association found by some studies.

However, other methodological factors may lie behind the lack of a positive association. No study among those selected, considered and adjusted for dietary patterns. Evaluation of dietary patterns avoid potential confounding with other aspects of the diet, increase the ability to evaluate stronger effects due to the cumulative effects of many dietary characteristics, and allow for evaluation of the interaction between synergistic components [51, 52]. A simplified approach that focuses only on UPF consumption fails to consider the substitution effects of foods and associated foods [53]. In fact, foods are complex combinations of nutrients and other compounds that act synergistically within and between food combinations: UPFs’ consumption in a various diet that is balanced in terms of calories and nutrients may not have the same effect when consumed in a dietary pattern that is high in calories and whose consumption of UPFs leads to the reduction of foods of higher nutritional value. In fact, as reported by Stewart et al. [54], consumption of foods typical of the UPF-rich Western diet was not found to be related to an increased risk of cardiovascular disease when included in a Mediterranean dietary pattern. It resulted more important to eat foods typical of a healthy dietary pattern than to avoid foods typical of the Western diet. Total energy intake and physical activity are defining factors of energy balance, and may, therefore, affect the association between UPF consumption and obesity and adiposity parameters. Of the articles included in this review, six made adjustments for physical activity level [35, 37, 39, 42–44] and four controlled for total energy intake [39, 40, 42, 43]. Less than a third of studies (3 out of 10) controlled for both energy intake and expenditure [39, 42, 43], with the consumption of UPF remaining positively associated with the excess of body weight and fat in two of them [42, 43]. This suggests that the impact of UPF on measures of body weight and fat is mediated by their caloric value and also by other factors, such as nutritional quality. In a mediation analysis, Costa et al. [42] observed that 58% (95% CI 0.05, 0.10) of the total effect of UPF consumption over the change in FMI from 6 to 11 years of age, was mediated by the caloric value of UPF. The remaining 42% could, at least in part, due to the direct effect of the UPF (some of it was presumably the effect of variables that were not considered). Since body weight and fat are strongly related to total caloric intake, the use of a nutrient density model (e.g., %UPF) without further inclusion of total energy intake among the covariates was not considered sufficient to control for confounding by total energy intake, as also suggested by Willett et al. [55]. Only one study accounted for underreporting of dietary intake [37], a well-known problem in nutritional epidemiology. Dietary underreporting has been found frequent in adolescents and associated with weight status [56]. There is thus a likelihood that overweight and obese adolescents underreported their dietary intake, flattening or reversing the association between UPF consumption and weight and fat outcomes. With regard to children, parents were responsible for reporting food intake on their behalf, and several studies showed that parents reliably reported their children’s food intake [57]. A further source of bias may be the method used for dietary assessment. Three of the four studies that found a positive association with weight and fat outcomes used a 24-h recall repeated on multiple occasions or a food diary [38, 43, 44], while only one used an FFQ [42]. Although the FFQ is the method most commonly used in epidemiological studies, it contains a substantial amount of measurement error. Many details of food intake are not measured, and quantification of intake is not as accurate as with the 24-h recall or food diary. Inaccuracies arise from an incomplete list of all possible foods and from errors in frequency and estimates of usual portion sizes [58]. The non-standardization by sex and age of obesity and adiposity measures, which naturally change with age, may be another reason for the lack of association between weight and fat measures and dietary habits, leading to a misinterpretation of the relationship. Almost all studies using non-standardized measures found no or inverse association, whereas most studies finding a positive association used *z* scores or height-standardized indices. Moreover, the methods used for body fat assessment, the use of a non-gold-standard method may be the reason for finding a non-positive association between UPF consumption and body fat. Fat mass by bioelectrical impedance was found to be inversely associated with UPF consumption in one study [37]. In contrast, in two out of three studies using BODPOD and DEXA, higher fat mass, expressed as both total body fat percentage and Fat Mass Index, was associated with a greater UPF consumption [42, 43].

Among the limitations of the present review, half of the studies had a cross-sectional design, which by its nature does not allow for the identification of a causal link between the exposure and the outcome. Moreover, it must be also considered that dietary habits at the time of recruitment may not reflect the past diet that led to the current weight status and body composition. On the other hand, it should be emphasized that dietary habits may change during the different stages of childhood and adolescence. More longitudinal studies with repeated measurements of food intake are needed to have a better representation of habitual diet and identify the direction of its relationship with the parameters of obesity and adiposity. An additional limitation is the identification of confounders judged to be far from optimal, as shown in Fig. 2, in three cross-sectional [36, 40, 41] and one longitudinal [38] study. In these studies, important confounders such as energy expenditure (or physical activity) were not considered. Moreover, in a longitudinal study [37], the retention rate during follow-up was suboptimal (< 80%). Another limitation is narrow geographic representativeness. Seventy percent of the selected studies were conducted on samples of Brazilian children and adolescents, limiting the generalizability of these results to other countries. Although the NOVA classification is recognized by several international organizations and adapts well to the dietary habits of the country where it was developed (Brazil), it is indeed important to consider that the classification may not be adequate in a cultural context with different dietary habits. A recent study described the nutritional composition of UPFs marketed in Italy, finding that a significant proportion of foods (23%) were considered of high nutritional quality considering three front-of-pack labeling schemes [59]. Therefore, further studies conducted on different populations are required for the development of nutritional policies and recommendations.

Nevertheless, this review also has some strengths. We are not aware of previous systematic reviews on the association between ultra-processed foods according to NOVA classification and obesity and adiposity parameters in children and adolescents. Selecting only studies with a common exposure classification reduces variability among studies.

Summarizing, the evidence for an association between consumption of ultra-processed foods and obesity and adiposity in children and adolescents is limited and heterogeneous. Further rigorously performed studies that address limitations and disparities among studies are needed to more clearly investigate and define these associations. Specifically, key questions remain unanswered and will require further investigation in future: (1) assessment of UPF consumption by adjusting for dietary pattern to consider the effects of food substitution and associated foods; (2) studies with longitudinal design with repeated measures of food intake for identification of a causal link between UPF consumption and childhood obesity and adiposity; (3) multicenter studies including multiple countries to overcome the low geographic representativeness of the current literature and to allow generalizability of these findings to other countries.

## Conclusion

Studies currently available in the literature report conflicting data about the association between consumption of ultra-processed foods and obesity and adiposity parameters among children and adolescents, probably due to methodological limitations. However, longitudinal studies with long follow-up provide some level of consistency in supporting the relationship between high consumption of ultra-processed foods and greater whole-body and abdominal adiposity. Further well-designed studies addressing the limitations reported in the present review are required to clearly define the association between the consumption of ultra-processed foods and obesity in children and adolescents.

## Data Availability

Not applicable.
